# Muscle mass and muscle strength are associated with pre- and post-hospitalization falls in older male inpatients: a longitudinal cohort study

**DOI:** 10.1186/s12877-018-0812-5

**Published:** 2018-05-16

**Authors:** Jeanine M. Van Ancum, Mirjam Pijnappels, Nini H. Jonkman, Kira Scheerman, Sjors Verlaan, Carel G. M. Meskers, Andrea B. Maier

**Affiliations:** 1 Department of Human Movement Sciences, @AgeAmsterdam, Vrije Universiteit Amsterdam, Amsterdam Movement Sciences, Amsterdam, the Netherlands; 2 Department of Internal Medicine, Section of Gerontology and Geriatrics, VU University Medical Center, Amsterdam Movement Sciences, Amsterdam, the Netherlands; 3 Department of Rehabilitation Medicine, VU University Medical Center, Amsterdam Movement Sciences, Amsterdam, the Netherlands; 4 Department of Medicine and Aged Care, @AgeMelbourne, Royal Melbourne Hospital, University of Melbourne, Clinical Sciences Building, Royal Parade, Parkville Victoria, Melbourne, VIC 3010 Australia

**Keywords:** Accidental Falls, Aged, Hospitalization, Muscle strength, Sarcopenia

## Abstract

**Background:**

Low muscle mass and strength are highly prevalent in inpatients. It is acknowledged that low muscle mass and strength are associated with falls in community-dwelling older adults, but it is unknown if these muscle measures are also associated with falls in a population of older inpatients. This study aimed to investigate the association between muscle measures and pre- and post-hospitalization falls in older inpatients.

**Methods:**

An inception cohort of patients aged 70 years and older, admitted to an academic teaching hospital, was included in this study. Muscle mass and hand grip strength were measured at admission using bioelectrical impedance analysis and handheld dynamometry. Pre-hospitalization falls were dichotomized as having had at least one fall in the six months prior to admission. Post-hospitalization falls were dichotomized as having had at least one fall during the three months after discharge. Associations were analysed with logistic regression analysis.

**Results:**

The study cohort comprised 378 inpatients (mean age, SD: 79.7, 6.4 years). Fifty per cent of female and 41% of male patients reported at least one fall prior to hospitalization. Post-hospitalization, 18% of female and 23% of male patients reported at least one fall. Lower muscle mass was associated with post-hospitalization falls, and lower hand grip strength was associated with both pre- and post-hospitalization falls in male, but not in female, patients.

**Conclusions:**

These findings confirm the likely involvement of muscle mass and strength in the occurrence of pre- and post-hospitalization falls in a population of older inpatients, but only in males.

**Electronic supplementary material:**

The online version of this article (10.1186/s12877-018-0812-5) contains supplementary material, which is available to authorized users.

## Background

Sarcopenia, a combination of low muscle mass and low muscle strength [1], is prevalent in up to 25% of older inpatients at admission [2]. During hospitalization, low muscle mass and low muscle strength are associated with a higher incidence of adverse clinical events, malnutrition, a longer length of hospital stay, incomplete functional recovery and in-hospital mortality [2–6]. After discharge, low muscle mass and strength at admission have been associated with low cognitive function, depressive symptoms, poor quality of life, nursing home institutionalization, hospital readmission and mortality [2, 4, 5, 7–10].

In community-dwelling older adults, low muscle mass and low muscle strength, indicated by low leg extension force as well as low hand grip strength (HGS), have been associated with a higher risk of falls [11–16]. Intervention studies have shown that improving muscle mass and strength in older community-dwelling adults reduces the risk of falls [17, 18]. However, currently there is no evidence demonstrating that strength training as mono-therapy reduces the risk or rate of falling. In older patients with a previous hospitalization, a fall rate of 15% within the first month after discharge was reported [19]. Risk factors of post-discharge falls in older patients include male gender, depressed mood, reliance on an assistive device, decline in mobility during hospitalization and cognitive impairment at discharge [20, 21]. Low muscle mass and strength may be the underlying cause of most of these risk factors and should therefore conceptually be associated with falls.

We aimed to investigate whether muscle mass and strength are associated with pre- and post-hospitalization falls in older inpatients.

## Methods

### Study design

**Fig. 1:**
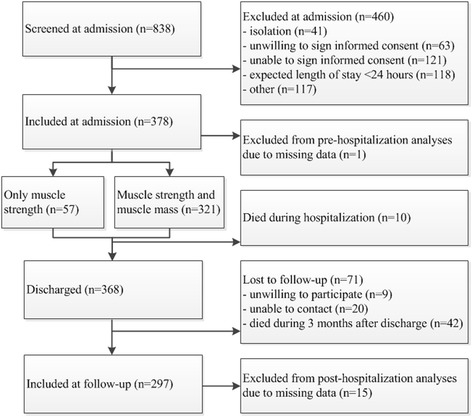
Flowchart on the number of patients included for each assessment

The Evaluation of Muscle parameters in a Prospective cohort of Older patients at clinical Wards Exploring Relations with bed rest and malnutrition (EMPOWER) study was a prospective, inception cohort study conducted from April until December 2015 at the VU university medical center in Amsterdam, the Netherlands. An extended description of the protocol is published elsewhere [22]. In short, patients aged 70 years and older, either electively or acutely admitted to the internal medicine, acute admission, trauma or orthopaedic ward, and who were hospitalized for > 24 h were included in the study. Patients were assessed within 48 h after admission (*n* = 378) and three months after discharge by a telephone interview (*n* = 297) (see Fig. 1). Main reason for loss to follow-up was death in the three months following hospitalization (*n* = 42). The study was approved by the Medical Ethics Committee of the VU University Medical Center and all included patients signed written informed consent.

### Clinical and demographic measures

Baseline characteristics were obtained from medical charts augmented with a bedside interview. Measures included: use of walking aid (yes/no), living independently (yes/no), weight (in kilograms, kg, using a weighting chair), height (in centimetres, cm, using knee height as proposed by the Longitudinal Aging Study Amsterdam, LASA formula [Male: 74,48 + (2,03 * knee height) - (0,15 * age), female: 68,74 + (2,07 * knee height) - (0,16 * age)]), current smoking (yes/no), current alcohol use (yes/no), type of admission (elective/acute), treating specialism (surgical including vascular and orthopaedic surgery/non-surgical including internal medicine, cardiology and neurology), number of chronic medications (polypharmacy defined as > 4 chronic medications), number of chronic diseases (comorbidities defined as > 1 chronic disease), disability (Katz-Activities of Daily Living, ADL [23]), pain (Numeric Rating Scale, NRS [24]), mobility (Functional Ambulatory Categories, FAC [25]), cognition (six item Cognitive Impairment Test, 6-CIT [26]), malnutrition (Short Nutritional Assessment Questionnaire, SNAQ [27]).

### Muscle measures

At admission, patients were assessed for muscle mass and muscle strength. Muscle mass was measured using direct segmental multi-frequency bioelectrical impedance analysis (DSM-BIA, InBody S10, Biospace Co., Ltd., Seoul) resulting in the following parameters: 1) absolute skeletal muscle mass (SMM); 2) skeletal muscle mass index (SMI), calculated as [SMM/height^2^] [1]; and 3) relative muscle mass (RMM), calculated as [SMM/weight] [28]. DSM-BIA could not be performed in patients with a pacemaker or an implantable cardioverter-defibrillator, plasters or bandages that could not be removed from the positioning place of the electrodes, or amputated arm and/or leg (*n* = 57, see Fig. 1).

HGS was measured using a hydraulic handheld dynamometer (Jamar, Sammons Preston Rolyan, IL, USA) sitting upright in a chair without support of the elbows. In case the patient was unable to get out of bed, HGS was measured with the bed in an angle of 30 degrees and the elbows unsupported. Repeated measurements were performed in the same position with two attempts per hand [29]. The maximum score out of four attempts was used for analysis.

### Pre- and post-hospitalization falls

A fall was defined as an event that resulted in “unintentionally coming to the ground or some lower level and other than as a consequence of sustaining a violent blow, loss of consciousness, sudden onset of paralysis as in stroke or an epileptic seizure” [30]. Pre-hospitalization falls were retrospectively assessed at admission with a questionnaire and dichotomized as at least one fall within six months before admission [31]. Post-hospitalization falls were prospectively assessed three months after discharge by a telephone interview and defined as at least one fall within the three months after discharge.

### Statistical analysis

Data are presented as mean ± standard deviation (SD) for normally distributed data, median and interquartile range (IQR) for skewed data and numbers and % for categorical data. Due to missing data, respectively one patient and 15 patients were excluded from the analyses on pre- and post-hospitalization falls.

All analyses were stratified for sex. The associations of muscle measures with pre-hospitalization and with post-hospitalization falls were investigated using logistic regression analyses. We first performed unadjusted analyses and subsequently adjusted for age, comorbidities, and height (only for HGS) or weight (only for RMM) in multivariable models. Both age and number of comorbidities were selected as covariates because of their association with muscle measures and fall risk [16, 32]. We added height to the model of HGS [33] and body weight to the model of RMM [34] as they effect these variables next to the risk of falls [35, 36]. To be able to compare the effect sizes of the different muscle measures, we performed post-hoc analyses and repeated the adjusted models using sex specific z-scores. Results were presented as odds ratios (OR) with 95% confidence intervals (CI) and *p*-values. Significance level was set at α = 0.05. Statistical Package for the Social Sciences (IBM SPSS Statistics for Windows, Version 23.0. Armonk, NY, IBM Corp) was used for all analyses.

## Results

Characteristics of the patients at baseline and at follow-up are shown in Table 1. Mean age (SD) was 79.7 (6.4) years of the entire patient cohort, 49% were female and 91% were living independently before hospitalization. In the six months before hospitalization, 50% of female and 41% of male patients experienced a fall. In the three months after discharge, 18% of female and 23% of male patients experienced a fall.

**Table 1:** Patient characteristics at admission and three months follow-up, stratified by sex

Characteristics	Included at baseline (*n* = 378)		Included at follow-up (*n* = 297)	
	♂ (*n* = 192)	♀ (*n* = 186)	♂ (*n* = 141)	♀ (*n* = 156)
Age, years, mean (SD)	79.1 (6.3)	80.3 (6.5)	78.1 (5.5)	80.3 (6.5)
Use of walking aid	92 (48.2)	108 (58.7)	64 (45.7)	91 (59.1)
Living independently	178 (92.7)	161 (89.0)	132 (93.6)	135 (88.2)
Weight, kg, mean (SD)	77.3 (15.4)	68.3 (16.7)	79.8 (15.2)	68.9 (17.3)
Height, cm, mean (SD)	175 (6.7)	161 (5.7)	176 (6.4)	161 (5.7)
BMI, kg/m^2^, mean (SD)	25.2 (4.5)	26.3 (6.3)	25.9 (4.4)	26.5 (6.5)
Current smoking	27 (14.5)	13 (7.1)	19 (13.8)	8 (5.2)
Alcohol use	91 (48.9)	55 (30.4)	80 (58.0)	45 (29.6)
Elective admission	25 (13.0)	33 (17.7)	23 (16.3)	29 (18.6)
Surgical specialism	76 (39.6)	95 (51.1)	65 (46.1)	83 (53.2)
Polypharmacy^a^	127 (66.1)	107 (57.5)	88 (62.4)	91 (58.3)
Comorbidities^b^	170 (88.5)	163 (88.6)	120 (85.1)	137 (89.0)
ADL-score, median (IQR)	0 (0–3)	1 (0–3)	0 (0–2)	1 (0–3)
NRS-score, median (IQR)	1 (0–4)	3 (0–6)	1 (0–4)	3 (0–6)
FAC-score, median (IQR)	2 (1–5)	2 (0–4)	3 (1–5)	2 (0–5)
6-CIT-score, median (IQR)	4 (0–8)	4 (1–10)	4 (0–8)	4 (0–10)
SNAQ-score, median (IQR)	0 (0–3)	1 (0–2)	0 (0–2)	0 (0–2)
LOS, days, median (IQR)	5 (3–7)	5 (3–9)	5 (2–7)	5 (3–9)
Pre-hospitalization falls^c^	79 (41.1)	93 (50.3)	57 (40.4)	79 (51.0)
Post-hospitalization falls^d^			32 (23.4)	26 (17.9)

All variables are presented as n (%), unless otherwise indicated

*BMI* Body Mass Index. *SNAQ* Short Nutritional Assessment Questionnaire. *ADL* KATZ Activities of Daily Living. *6-CIT* 6-item Cognitive Impairment Test. *NRS* Numerical Rating Scale for pain. *FAC* Functional Ambulation Categories. *LOS* Length of Stay. *SD* Standard Deviation. *IQR* Interquartile Range ^a^Number of medications > 4. ^b^Number of comorbidities > 1. ^c^Pre-hospitalization falls: Patients who reported at least one fall within 6 months before admission. ^d^Post-hospitalization falls (♂*n* = 137, ♀*n* = 145): Patients who reported at least one fall within 3 months after discharge

Table 2 shows the associations of muscle mass and HGS with pre-hospitalization falls. In male patients, lower HGS (OR, 95% CI, 0.94, 0.90–0.98) was associated with pre-hospitalization falls. For lower SMM and SMI a trend was observed (ORs, 95% CI, SMM: 0.94, 0.88–1.01 and SMI: 0.80, 0.64–1.01), but not for RMM (OR, 95% CI, 0.97, 0.90–1.04). In female patients, lower HGS was associated with pre-hospitalization falls, but this did not reach statistical significance (OR, 95% CI, 0.94, 0.88–1.00). No association was found between muscle mass and pre-hospitalization falls in female patients (ORs, 95% CI, SMM: 1.04, 0.95–1.13, SMI: 1.10, 0.85–1.42 and RMM: 0.99, 0.92–1.07).

**Table 2:** Muscle parameters at admission and pre- and post-hospitalization falls, stratified by sex

		*N*	Falls, Mean (SD)	No falls, Mean (SD)	Crude		Adjusted	
					OR (95% CI)	*p*-value	OR (95% CI)	*p*-value
Pre-hospitalization falls^a^								
HGS, kg	♂	192	23.5 (10.8)	27.9 (8.82)	**0.95 (0.92, 0.98)**	**0.003**	**0.94 (0.90, 0.98)**	**0.003**
	♀	185	14.3 (5.96)	15.6 (5.25)	0.96 (0.91, 1.01)	0.128	0.94 (0.88, 1.00)	0.062
SMM, kg	♂	158	28.9 (5.22)	30.4 (5.84)	0.95 (0.90, 1.01)	0.104	0.94 (0.88, 1.01)	0.074
	♀	162	22.8 (4.01)	22.3 (3.50)	1.04 (0.86, 1.13)	0.352	1.04 (0.95, 1.13)	0.393
SMI, kg/m^2^	♂	158	9.45 (1.49)	9.88 (1.45)	0.82 (0.65, 1.02)	0.076	0.80 (0.64, 1.01)	0.063
	♀	162	8.73 (1.32)	8.51 (1.17)	1.11 (0.87, 1.43)	0.399	1.10 (0.85, 1.42)	0.470
RMM, %	♂	158	38.9 (5.56)	39.2 (4.60)	0.99 (0.93, 1.06)	0.748	0.97 (0.90, 1.04)	0.383
	♀	162	33.2 (6.04)	34.3 (5.26)	0.97 (0.91, 1.02)	0.226	0.99 (0.92, 1.07)	0.856
Post-hospitalization falls^b^								
HGS, kg	♂	137	23.4 (9.16)	29.6 (9.90)	**0.94 (0.90, 0.98)**	**0.004**	**0.93 (0.88, 0.99)**	**0.020**
	♀	145	14.7 (5.43)	15.1 (5.56)	0.99 (0.91, 1.07)	0.746	1.02 (0.92, 1.12)	0.771
SMM, kg	♂	116	27.3 (3.71)	31.8 (5.61)	**0.81 (0.71, 0.91)**	**0.001**	**0.80 (0.71, 0.92)**	**0.001**
	♀	125	22.1 (4.36)	23.0 (3.75)	0.94 (0.83, 1.07)	0.320	0.92 (0.81, 1.06)	0.243
SMI, kg/m^2^	♂	116	9.08 (1.15)	10.2 (1.41)	**0.53 (0.36, 0.77)**	**0.001**	**0.50 (0.33, 0.76)**	**0.001**
	♀	125	8.39 (1.55)	8.80 (1.16)	0.75 (0.50, 1.13)	0.166	0.73 (0.48, 1.10)	0.132
RMM, %	♂	116	38.2 (4.70)	39.4 (5.32)	0.96 (0.88, 1.04)	0.309	**0.85 (0.75, 0.96)**	**0.009**
	♀	125	32.9 (6.51)	33.7 (5.04)	0.97 (0.89, 1.06)	0.533	0.95 (0.85, 1.07)	0.435

*CI* Confidence Interval. *HGS* Hand Grip Strength. *OR* Odds Ratio. *SD* Standard Deviation. *SMI* Skeletal Muscle Index. *SMM* Skeletal Muscle Mass. *RMM* Relative Muscle Mass. *Adjusted model* adjusted for age, comorbidities, HGS for height and RMM for weight. Statistically significant results are presented in bold. ^a^Pre-hospitalization falls, yes: *N* = 172, no: *N* = 205. ^b^Post-hospitalization falls, yes: *N* = 58, no: *N* = 224

The associations of muscle mass and HGS with post-hospitalization falls are shown in Table 2. In male patients, lower HGS, SMM and SMI (ORs,95% CI, respectively: 0.93, 0.88–0.99, 0.80, 0.71–0.92 and 0.50, 0.33–0.76) were associated with post-hospitalization falls. Lower RMM was associated with falls after adjustment for confounders (OR, 95% CI, 0.85, 0.75–0.96). No association were found between muscle measures and post-hospitalization falls in female patients (ORs, 95% CI, HGS: 1.02, 0.92–1.12, SMM: 0.92, 0.81–1.06, SMI: 0.73, 0.48–1.10 and RMM: 0.95, 0.85–1.07).

The results of the post-hoc analyses with sex specific z-scores for comparison of the effect sizes between muscle measures are shown in Additional file 1: Table S1. Taking the significant associations into account, HGS had the largest effect size for pre- hospitalization falls, and SMM had the largest effect size for post-hospitalization falls in male patients.

## Discussion

In older male patients, lower absolute and relative muscle mass were associated with post-hospitalization falls, and lower HGS was associated with pre- and post-hospitalization falls. In female patients, no significant associations between muscle measures and pre- and post-hospitalization falls were found.

We found that absolute muscle mass was not associated with pre-hospitalization falls, but was associated with post-hospitalization falls, in male inpatients. The difference between pre- and post-hospitalization falls in the association with muscle mass is rather small, which might be due to differences in recall bias pre- and post-hospitalization. Our results are in line with a previous study of a large sample of community-dwelling older adults, in which people who had fallen in the previous year had significantly lower absolute muscle mass [16]. Furthermore, we found that RMM was significantly associated with post-hospitalization falls after adjustment for weight. This finding is in concordance with a previous study of community-dwelling older males, in which RMM was associated with risk of falls, after adjustment for fat mass [15]. SMM, SMI and RMM show different effect sizes, because of different scaling of the measures. Yet, comparing the effect sizes with z-scores indicates that all three measures are valuable determinants of post-hospitalization falls. Studies in older community-dwelling adults also showed an association of falls with upper and lower extremity weakness [13, 17]. Experimental studies with induced gait perturbations showed significantly lower limb muscle strength and lower HGS in fallers compared with non-fallers [14]. In line with our results, sarcopenia – defined using various definitions including absolute or relative muscle mass, isolated or combined with HGS and gait speed – was found to be associated with falls in community-dwelling older adults [11, 12].

In contrast to their male counterparts, female inpatients had relatively lower HGS and lower population variation. The HGS value of female inpatients is comparable with adults of the general population above 89 years of age [37] or those who are chronically ill [32]. The relatively lower strength and lower variation may account for the non-significant relation with either pre- or post-hospitalization. The contribution of other risk factors for falls, such as active disease, multi-morbidity and polypharmacy, may also be sex specific [38, 39]. None of the previous mentioned studies in community-dwelling adults reported their results separately for females, complicating the comparison to our results.

### Clinical implications and future research

Muscle strength and power are essential in the maintenance of balance. Also, a quick recovery during loss of balance, coupled with muscle strength, seem to exhibit a superior role over muscle mass in screening and intervening in fall prevention [17, 40]. However, an independent causality between muscle weakness and falls in older people has yet to be proven [41, 42]. The measured effect sizes of HGS and muscle mass are clinically meaningful, since an increase of one kg of muscle mass or strength leads to a large decrease in the odds of falls. These findings underline the potential for beneficial outcomes of interventions directed at increasing muscle strength and mass. Identification at an early stage in clinical practice of male inpatients at risk of falls, by use of relatively simple measures such as muscle mass and HGS, could allow for development of targeted interventions and potentially reduce healthcare costs in the long term. Despite the lack of significant associations with pre- and post-hospitalization falls in female patients, screening of female patients with low muscle mass and muscle strength at an early stage during hospitalization is important to identify females at risk of other detrimental outcomes [2].

### Strengths and limitations

To the best of our knowledge, this was the first study investigating the association between muscle measures and pre- and post-hospitalization falls in older inpatients. Selection bias was minimized through the study design as an inception cohort. An important limitation was the self-reporting of falls by patients, both in the questionnaire on admission and in the telephone interview three months after discharge. Self-reporting may be prone to recall bias, but this was unavoidable due to the study cohort design including predominantly acutely admitted patients. We did not record the number of falls per patient, since this is likely to be affected by recall bias. Furthermore, the dropout of patients between discharge from hospital and the telephone interview may have induced a selection bias, due to the death of more than half of the participants in this frail sample population.

## Conclusions

In this prospective, inception cohort study of inpatients aged 70 years and older, we found an association between post-hospitalization falls and muscle mass, and between pre- and post-hospitalization falls and HGS in male patients. No such association was found among female patients. Further research is needed to provide evidence on the causality of muscle mass and muscle strength in pre- and post-hospitalization falls in inpatients, for the development of inpatient interventions.

## Additional file

Additional file 1: **Table S1.** Z-scores of muscle parameters at admission and pre- and post-hospitalization falls, stratified by sex. Adjusted models of the standardized measures of HGS, SMM, SMI and RMM stratified for sex by z-scores. (DOCX 17 kb)

## Abbreviations

6-item CIT: 6-item Cognitive impairment test
CI: Confidence intervals
DSM-BIA: Direct segmental multi-frequency bioelectrical impedance analysis
EMPOWER: The evaluation of muscle parameters in a prospective cohort of older patients at clinical Wards Exploring Relations with bed rest and malnutrition
FAC: Functional ambulatory categories
HGS: Hand grip strength
IQR: Interquartile range
KATZ-ADL: Katz index of independence in activities of daily living
LASA: Longitudinal Aging Study Amsterdam
NRS: Numeric rating scale
OR: Odds ratios
RMM: Relative muscle mass
SD: Standard deviation
SMI: Skeletal muscle mass index
SMM: Skeletal muscle mass
SNAQ: Short Nutritional Assessment Questionnaire
SPSS: Statistical Package for the Social Sciences
