# Supplementary material for: Muscle mass and muscle strength are associated with pre- and post-hospitalization falls in older male inpatients: a longitudinal cohort study
Source: BMC Geriatr. 2018 May 16;18:116. doi: 10.1186/s12877-018-0812-5 (PMC5956790; doi:10.1186/s12877-018-0812-5)
Supplement: Supplementary file 1 — Table S1. Z-scores of muscle parameters at admission and pre- and post-hospitalization falls, stratified by sex. Adjusted models of the standardized measures of HGS, SMM, SMI and RMM stratified for sex by z-scores. (DOCX 17 kb) [file 12877_2018_812_MOESM1_ESM.docx]

| **Supplementary Table 1. Z-scores of muscle parameters at admission and pre- and post-hospitalization falls, stratified by sex.** | | | | |
| --- | --- | --- | --- | --- |
|  |  | N | Adjusted |  |
|  |  |  | OR (95% CI) | *p*-value |
| **Pre-hospitalization falls** * |  |  |  |  |
| HGS Z-score | ♂ | 192 | **0.53 (0.35, 0.80)** | **0.003** |
|  | ♀ | 185 | 0.70 (0.49, 1.02) | 0.062 |
| SMM Z-score | ♂ | 158 | 0.72 (0.50, 1.03) | 0.074 |
|  | ♀ | 162 | 1.15 (0.83, 1.60) | 0.393 |
| SMI Z-score | ♂ | 158 | 0.72 (0.51, 1.02) | 0.063 |
|  | ♀ | 162 | 1.13 (0.82, 1.55) | 0.470 |
| RMM Z-score | ♂ | 158 | 0.85 (0.60, 1.22) | 0.383 |
|  | ♀ | 162 | 0.96 (0.64, 1.45) | 0.856 |
| **Post-hospitalization falls** † |  |  |  |  |
| HGS Z-score | ♂ | 137 | **0.49 (0.27, 0.89)** | **0.020** |
|  | ♀ | 145 | 1.09 (0.62, 1.92) | 0.771 |
| SMM Z-score | ♂ | 116 | **0.29 (0.14, 0.61)** | **0.001** |
|  | ♀ | 125 | 0.74 (0.45, 1.22) | 0.243 |
| SMI Z-score | ♂ | 116 | **0.36 (0.19, 0.67)** | **0.001** |
|  | ♀ | 125 | 0.67 (0.40, 1.13) | 0.132 |
| RMM Z-score | ♂ | 116 | **0.44 (0.24, 0.82)** | **0.009** |
|  | ♀ | 125 | 0.77 (0.40, 1.49) | 0.435 |
| CI: Confidence Interval. HGS: Hand Grip Strength. OR: Odds Ratio. SD: Standard Deviation. SMI: Skeletal Muscle Index. SMM: Skeletal Muscle Mass. RMM: Relative Muscle Mass. Adjusted model: adjusted for age, comorbidities, HGS for height and RMM for weight. Statistically significant results are presented in bold. * Pre-hospitalization falls, yes: N=172, no: N=205. † Post-hospitalization falls, yes: N=58, no: N=224. | | | | |
